# Incidence of Recurrence and Time to Recurrence in Stage I to III Colorectal Cancer: A Nationwide Danish Cohort Study

**DOI:** 10.1001/jamaoncol.2023.5098

**Published:** 2023-11-16

**Authors:** Jesper Nors, Lene Hjerrild Iversen, Rune Erichsen, Kåre Andersson Gotschalck, Claus Lindbjerg Andersen

**Affiliations:** 1 Department of Molecular Medicine, Aarhus University Hospital, Aarhus, Denmark; 2 Department of Clinical Medicine, Aarhus University, Aarhus, Denmark; 3 Department of Surgery, Aarhus University Hospital, Aarhus, Denmark; 4 Department of Clinical Epidemiology, Aarhus University Hospital, Aarhus, Denmark; 5 Department of Surgery, Randers Regional Hospital, Randers, Denmark; 6 Department of Surgery, Horsens Regional Hospital, Horsens, Denmark

## Abstract

**Importance:**

Management of colorectal cancer (CRC) has been updated continuously over the past 2 decades. While the combination of these initiatives has had implications for improved survival, the implications for rates of recurrence remain unexplored.

**Objective:**

To ascertain the rates of recurrence and describe time to recurrence within 5 years of surgery with curative intent for stages I to III CRC.

**Design, Setting, and Participants:**

This cohort study used the Danish Colorectal Cancer Group Database to identify patients with Union for International Cancer Control (UICC) stages I to III CRC who underwent primary surgery between January 1, 2004, and December 31, 2019. They were followed up until recurrence (event), death (competing event), diagnosis of a second cancer (competing event), emigration (censoring event), 5 years postoperatively (censoring event), or January 1, 2023 (censoring event), whichever came first. Recurrence status was ascertained through individual-level linked data from the Danish Cancer Registry, Danish National Patient Registry, and Danish Pathology Registry using a validated algorithm. Data were analyzed from January 1 to August 8, 2023.

**Exposure:**

Primary surgery performed during 3 calendar periods (2004-2008, 2009-2013, and 2014-2019) stratified by tumor site (colon or rectum) and UICC stage (I, II, and III).

**Main Outcomes and Measures:**

Stage-specific 5-year recurrence reported as the cumulative incidence function (CIF) of recurrence, the association between calendar period of primary surgery and recurrence risk reported as subdistribution hazard ratios (sHRs), and the time from surgery to recurrence.

**Results:**

Of the 34 166 patients with UICC stages I to III CRC (median [IQR] age, 70 [62-77] years); 18 552 males [54.3%]) included in the study, 7027 developed recurrence within 5 years after the primary surgery. For colon cancer, the 5-year CIF of recurrence decreased over the 3 calendar periods from 16.3% to 6.8% for UICC stage I, from 21.9% to 11.6% for UICC stage II, and from 35.3% to 24.6% for UICC stage III colon cancer. For rectal cancer, the 5-year CIF decreased over the 3 periods from 19.9% to 9.5% for stage I, from 25.8% to 18.4% for stage II, and from 38.7% to 28.8% for stage III disease. Patients with stage III disease had a shorter time from surgery to recurrence compared with those with stage I disease (time ratio stage III vs stage I = 0.30; 95% CI, 0.28-0.32). Cancers detected through screening were associated with lower stage-adjusted risks of recurrence (sHR, 0.81; 95% CI, 0.73-0.91) compared with cancers not detected through screening.

**Conclusions and Relevance:**

In this cohort of patients with CRC, the risk of recurrence decreased in patients with stages I to III disease during the study period. Cancer detection by screening was associated with an even lower risk of recurrence. Time to recurrence differed according to UICC stage. Because the risk of recurrence was so low in selected patient groups, future research is warranted to explore risk-stratified surveillance protocols in patients with CRC.

## Key Points

**Question:**

What are the colorectal cancer (CRC) stage-specific rates of recurrence and time from surgery to recurrence in patients undergoing surgery with curative intent?

**Findings:**

In this cohort study of 34 166 patients with CRC who underwent surgery from 2004 to 2019, the risk of recurrence decreased over time, and higher disease stage was associated with shorter times from surgery to recurrence. Screening-detected CRC was associated with a lower risk of recurrence.

**Meaning:**

Findings of this study suggest that the risk of CRC recurrence and the time from surgery to recurrence differ between patient groups, highlighting the importance of further research on risk-stratified surveillance.

## Teaser

This cohort study investigates stage-specific rates and timing of colorectal cancer recurrence using data from Danish national health registries from 2004 to 2019.

## Introduction

Colorectal cancer (CRC) represents a considerable health burden worldwide.^1^ The standard of care for patients with nonmetastatic CRC (Union for International Cancer Control [UICC] TNM stages I-III) is surgery, with clinical staging informing the use of neoadjuvant therapy and histopathological staging informing the use of adjuvant chemotherapy.^2,3^ Although surgery is intended to be curative treatment, approximately 20% of patients experience recurrence of disease. Therefore, all patients with stages I to III CRC are offered postoperative surveillance.

Over the past 2 decades, several initiatives aimed at reducing the risk of recurrence and improving survival of patients with CRC have been implemented in most countries, including Denmark^4^ (eFigure 1 in Supplement 1). Initiatives include increased focus on improving primary treatment with regularly updated national recommendations,^5^ a multidisciplinary team approach,^6,7^ enhanced recovery after surgery protocols,^8^ and implementation of new surgical procedures; the latter include central vascular ligation and dissection along the embryological fascia as the underlying principle of complete mesocolic excision^9^ and total mesorectal excision,^10^ with increased lymph node yield. In addition, the treatment of CRC has been centralized to specialized centers,^11^ and the pathological examinations of surgical specimens^12,13^ and postoperative surveillance have been standardized.^14,15,16,17^ Lastly, population-based screening programs have been implemented in several countries,^18^ identifying patients with asymptomatic CRC.^19^ While most of the initiatives have been associated with better outcomes individually, nationwide population-based studies are needed to investigate the implications of these initiatives for the risk of recurrence.

The aim of this study was to determine colon and rectal cancer stage-specific rates of recurrence and, for those with recurrence, the time from surgery to recurrence (TSTR) in a nationwide cohort of patients who underwent surgery with curative intent for nonmetastatic CRC from 2004 to 2019 by comparing 3 calendar periods of surgery with curative intent: 2004 to 2008, 2009 to 2013, and 2014 to 2019. A secondary aim was to describe the risk of recurrence in colon and rectal cancers detected through screening.

## Methods

### Design and Study Population

This nationwide registry-based cohort study included all patients who underwent surgery with curative intent for UICC TNM stages I to III CRC between January 1, 2004, and December 31, 2019. The study was approved by the Danish Colorectal Cancer Group (DCCG) and the Danish Data Protection Agency (Central Denmark Region) and adhered to the European Union General Data Protection Regulations.^20^ The study was based on anonymized registry data and therefore was exempt from review and informed consent in accordance with the General Data Protection Regulations. The study was reported according to the Strengthening the Reporting of Observational Studies in Epidemiology (STROBE) reporting guideline.^21^

Patients were identified through the DCCG Database, which is a prospective national clinical quality database with information on all patients treated for first-time CRC in Denmark.^22^ A timeline and description of the management of nonmetastatic CRC in Denmark from 2004 to 2019 are provided in eFigure 1 and the eMethods in Supplement 1.

Patients with a diagnosis of cancer other than CRC or nonmelanoma skin cancer (NMSC) prior to curative CRC surgery were excluded. Patients were also excluded if they emigrated, died, or were diagnosed with a new primary cancer other than CRC or NMSC or metastasis of unspecified origin within 180 days of the index CRC surgery.

Patients were followed up from 180 days after surgery until the following event (whichever came first): recurrence (event), second primary cancer (competing event), death (competing event), emigration (censoring event), 5 years postoperatively (censoring event), or January 1, 2023 (censoring event), when prospectively collected data from the national health care registries were retrieved (eTable 1 in Supplement 1). Hence, patients undergoing curative surgery from 2004 to 2017 had 5 years of follow-up, those undergoing curative surgery in 2018 had 4 years of follow-up, and those undergoing curative surgery in 2019 had 3 years of follow-up.

### Data Sources and Identification of Recurrence

Individual-level data were obtained from Danish health care registries,^23^ including the Danish Cancer Register (DCR),^24^ the Danish National Patient Registry (DNPR),^25^ and the Danish Pathology Registry (DPR).^26^ Data records were linked using the unique 10-digit civil registration number issued to each Danish resident by the Danish Civil Registration System.^27^

The DCCG Database provided information on the date of CRC diagnosis, date of surgery, and patient characteristics, including participation in the National Danish Colorectal Cancer Screening Program, which started in 2014 (linked from the Danish Colorectal Cancer Screening Database^28^). The DNPR contains administrative and clinical data and provides information on chemotherapeutic treatment and diagnosis of metastases. The DCR contains data on the incidence of malignant neoplasms. The DPR provides electronically recorded data on all biological specimens according to national guidelines for uniform registration using the Danish version of the Systematized Nomenclature of Medicine (SNOMED) codes.^29^

Recurrence was detected using a previously described algorithm,^30^ with a few optimizations^31^ (eFigure 2 in Supplement 1). The algorithm defines recurrence when at least 1 of the following 4 indicators occurs:

1. Metastasis code (*International Statistical Classification of Diseases and Related Health Problems, Tenth Revision* [*ICD-10*] DC76-80) present in the DNPR or the DCR without a new primary cancer diagnosis other than CRC (*ICD-10* code DC18 or DC20) or NMSC (*ICD-10* code DC44) in the DNPR or the DCR;
2. Cytostatic therapy codes (BWHA1-2, BOHJ17, or BOHJ19B1) present in the DNPR 60 or more days after the last cytostatic therapy code and registered by an oncological department (eTable 2 in Supplement 1) without a new primary cancer diagnosis other than CRC or NMSC in the DNPR or the DCR;
3. SNOMED combinations present in the DPR; or
4. DNPR codes specific to CRC local recurrence (DC189X, DC209X, or DC991).

All 4 indicators require the code to be registered 180 or more days after surgery.

### Covariates

The dates of curative surgery were grouped into 3 calendar periods: 2004 to 2008, 2009 to 2013, and 2014 to 2019. The latter period coincides with the introduction of the National Danish Colorectal Cancer Screening Program in 2014.

Eligible patients were categorized by sex (male or female), age group at curative surgery (<55, 55-64, 65-74, 75-84, or ≥85 years), pathological UICC stage (I, II, or III), Charlson Comorbidity Index score (0, 1-2, or ≥3; higher scores indicated a higher number of comorbidities),^32^ site of CRC tumor (colon or rectum), and surgical priority (elective or emergency). Disease stages were categorized according to the fifth edition^33^ of the UICC TNM classification from 2004 to 2016 and the eighth edition^34^ from 2017 to 2019. Patients in the 2014 to 2019 period were categorized according to whether the cancer was detected by screening (screening detected) or not (nonscreening detected).

### Statistical Analysis

Patient demographics and treatment characteristics are presented with continuous data as medians (IQRs) and with categorical variables as the total number and percentage. Multivariable analysis was performed to investigate the association between calendar period of curative surgery and the risk of CRC recurrence, adjusted for patient and treatment characteristics. Risk of recurrence was calculated as 1-, 3-, and 5-year cumulative incidence function (CIF) estimates, with second primary cancer and death treated as competing events. Cumulative incidence function curves were constructed using the Aalen-Johansen estimator for visualization, grouped by the calendar period of curative surgery, and stratified by tumor site and UICC TNM stage. The association between calendar period of curative surgery and the risk of recurrence was estimated using the Fine-Gray competing risks method, with second primary cancer and death treated as competing events, and was reported as subdistribution hazard ratios (sHRs) with 95% CIs.^35^ Tumor site– and stage-specific sHRs were adjusted for age, sex, and Charlson Comorbidity Index score.

For patients with CRC recurrence, the TSTR was calculated as the time between the date of curative CRC surgery and the date on which at least 1 of the 4 indicators in the algorithm defined recurrence. The difference in TSTR between UICC stages was estimated as a time ratio (with 95% CIs) using an accelerated failure time model including tumor site, age, sex, and Charlson Comorbidity Index score and right-censoring data for patients at the time of a second primary cancer, death, emigration, 5 years postoperatively, or January 1, 2023, whichever came first.

A subgroup analysis was performed to assess the association between patients with screening-detected CRC and those with nonscreening-detected CRC. The risk of recurrence was calculated and 1-, 3-, and 5-year CIF estimates of recurrence and sHRs adjusted for age, sex, and Charlson Comorbidity Index score were reported.

Statistical analyses were carried out using RStudio, version 2021.9.1.372 (Posit PBC). Data were analyzed from January 1 to August 8, 2023.

## Results

We identified 45 274 patients who underwent curative surgery for pathological UICC stages I to III CRC between 2004 and 2019 (eFigure 3 in Supplement 1). After applying exclusion criteria (approximately 15% of patients excluded; eResults in Supplement 1), the final cohort consisted of 34 166 patients (median [IQR] age, 70 [62-77] years; 18 552 males [54.3%] and 15 614 females [45.7%]), with 9135 patients who underwent curative surgery between 2004 and 2008, 9780 between 2009 and 2013, and 15 251 between 2014 and 2019.

Demographic and clinical characteristics of patients with colon or rectal cancer stratified by calendar period of surgery are summarized in Table 1. From 2004 to 2019, the distribution of UICC stages shifted toward a higher proportion of stage I CRC and a lower proportion of stage II CRC (eFigure 4 in Supplement 1). The shift was most prominent from 2014 to 2019, consistent with implementation of a population-wide CRC screening program in 2014.

**Table 1.** Demographics and Treatment Characteristics of Patients With Stages I to III Colorectal Cancer Grouped by Tumor Site and Stratified by Calendar Period of Primary Surgery

Variable	Overall No. (%) (N = 34 166)	Patients with colon cancer, No. (%)			Patients with rectal cancer, No. (%)		
		2004-2008 (n = 5805)	2009-2013 (n = 6411)	2014-2019 (n = 10 470)	2004-2008 (n = 3330)	2009-2013 (n = 3369)	2014-2019 (n = 4781)
Sex							
Female	15 614 (45.7)	2959 (51.0)	3231 (50.4)	4961 (47.4)	1319 (39.6)	1284 (38.1)	1860 (38.9)
Male	18 552 (54.3)	2846 (49.0)	3180 (49.6)	5509 (52.6)	2011 (60.4)	2085 (61.9)	2921 (61.1)
Age, median (IQR), y	70 (62-77)	71 (63-78)	71 (64-79)	71 (64-77)	67 (59-75)	68 (61-75)	68 (60-74)
Body mass index, median (IQR)^a^	25.5 (23.0-28.7)	25.0 (22.6-27.8)	25.2 (22.6-28.4)	25.9 (23.2-29.2)	25.3 (22.9-27.9)	25.4 (23.1-28.4)	25.7 (23.3-28.7)
Charlson Comorbidity Index score							
0	20 607 (60.3)	3635 (62.6)	3918 (61.1)	5710 (54.5)	2243 (67.4)	2306 (68.4)	2795 (58.5)
1-2	10 712 (31.4)	1734 (29.9)	1982 (30.9)	3622 (34.6)	901 (27.1)	879 (26.1)	1594 (33.3)
≥3	2847 (8.3)	436 (7.5)	511 (8.0)	1138 (10.9)	186 (5.6)	184 (5.5)	392 (8.2)
Place of residence							
Region of Northern Denmark	3825 (11.2)	667 (11.5)	681 (10.6)	1194 (11.4)	368 (11.1)	388 (11.5)	527 (11.0)
Central Denmark Region	7321 (21.4)	1151 (19.8)	1326 (20.7)	2359 (22.5)	706 (21.2)	705 (20.9)	1074 (22.5)
Region of Southern Denmark	7947 (23.3)	1323 (22.8)	1533 (23.9)	2436 (23.2)	782 (23.5)	807 (24.0)	1066 (22.3)
Region Zealand	5602 (16.4)	937 (16.1)	1132 (17.7)	1701 (16.2)	525 (15.8)	547 (16.2)	760 (15.9)
Capital Region of Denmark	9471 (27.7)	1727 (29.8)	1739 (27.1)	2780 (26.6)	949 (28.5)	922 (27.4)	1354 (28.3)
UICC stage							
I	8664 (25.4)	1004 (17.3)	1145 (17.9)	2885 (27.6)	799 (24.0)	939 (27.9)	1892 (39.6)
II	13 755 (40.3)	2823 (48.6)	3155 (49.2)	4080 (39.0)	1238 (37.2)	1165 (34.6)	1294 (27.1)
III	11 747 (34.4)	1978 (34.1)	2111 (31.9)	3505 (33.5)	1293 (38.8)	1265 (37.5)	1595 (33.4)
T category^b^							
T1	3890 (12.6)	456 (7.9)	444 (7.1)	1679 (16.5)	237 (10.6)	240 (9.5)	834 (21.7)
T2	5137 (16.6)	607 (10.5)	691 (11.0)	1502 (14.8)	545 (24.4)	652 (25.8)	1140 (29.6)
T3	16 567 (53.7)	3630 (62.5)	3670 (58.4)	5188 (51.0)	1173 (52.6)	1294 (51.2)	1612 (41.9)
T4	3806 (12.3)	812 (14.0)	1001 (15.9)	1493 (14.7)	147 (6.6)	173 (6.8)	180 (4.7)
Tx	1456 (4.7)	300 (5.2)	477 (7.6)	303 (3.0)	129 (5.8)	167 (6.6)	80 (2.1)
N category^c^							
N0	18 547 (60.3)	3430 (59.6)	3705 (59.1)	6346 (62.4)	1256 (56.7)	1428 (56.7)	2382 (62.0)
N1	6351 (20.6)	1226 (21.3)	1243 (19.8)	2169 (21.3)	431 (19.5)	482 (19.1)	800 (20.8)
N2	3533 (11.5)	708 (12.3)	730 (11.6)	1111 (10.9)	280 (12.6)	327 (13.0)	377 (9.8)
Nx	2338 (7.6)	390 (6.8)	596 (9.5)	538 (5.3)	248 (11.2)	282 (11.1)	284 (7.4)
Histological classification							
Adenocarcinoma	31 150 (91.5)	5200 (90.0)	5703 (89.4)	9361 (89.7)	3161 (95.2)	3190 (94.8)	4535 (95.0)
Mucinous adenocarcinoma	2720 (8.0)	538 (9.3)	633 (9.9)	1015 (9.7)	146 (4.4)	161 (4.8)	227 (4.8)
Signet ring cell carcinoma	179 (0.5)	38 (0.7)	44 (0.7)	56 (0.5)	15 (0.5)	15 (0.4)	11 (0.2)
Adjuvant chemotherapy	8823 (25.8)	1392 (24.0)	2022 (31.5)	3076 (29.4)	337 (10.1)	869 (25.8)	1127 (23.6)
Neoadjuvant treatment	3310 (9.7)	0 (0)	128 (2.0)	305 (2.9)	1099 (33.0)	843 (25.0)	935 (19.6)
Priority of surgery^c^							
Elective	32 055 (94.0)	5107 (88.0)	5776 (90.1)	9796 (93.6)	3302 (99.2)	3348 (99.4)	4726 (99.7)
Emergency	2063 (6.0)	697 (12.0)	634 (9.9)	667 (6.4)	28 (0.8)	21 (0.6)	16 (0.3)

Abbreviation: UICC, Union for International Cancer Control.

^a^ Body mass index calculated as weight in kilograms divided by height in meters squared.

^b^ Not reported for patients treated with neoadjuvant oncological therapy.

^c^ If the patient underwent resection.

### Risk of Recurrence

Within 5 years after curative surgery, 7027 patients developed recurrence. The 5-year CIF of recurrence for CRC was 26.9% (95% CI, 26.0%-27.8%) in the 2004 to 2008 period, 22.2% (95% CI, 24.4%-23.0%) in the 2009 to 2013 period, and 15.8% (95% CI, 15.2%-16.4%) in the 2014 to 2019 period (eTable 3 in Supplement 1). Compared with the 2004 to 2008 period, the adjusted sHR in the 2009 to 2013 period was 0.82 (95% CI, 0.78-0.87), and the adjusted sHR in the 2014 to 2019 period was 0.59 (95% CI, 0.56-0.62).

**Figure 1.**
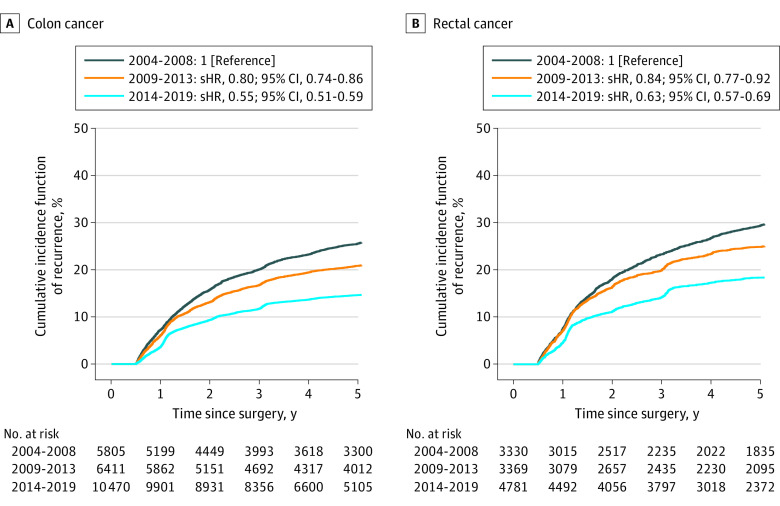
Cumulative Incidence Function Curves for Colorectal Cancer Recurrence Stratified by Tumor Site and Compared by Calendar Period of Curative Surgery Curves were constructed using the Aalen-Johansen estimator. Patients were right-censored at emigration, 5 years of follow-up, or on January 1, 2023, whichever came first. sHR indicates subdistribution hazard ratio calculated using Fine-Gray regression adjusted for sex, age, comorbidities, and Union for International Cancer Control stage.

For colon cancer, the 5-year CIF of recurrence decreased over time: from 25.5% (95% CI, 24.4%-26.6%) in the 2004 to 2008 period, to 20.8% (95% CI, 19.8%-21.8%) in the 2009 to 2013 period, and to 14.6% (95% CI, 13.9%-15.3%) in the 2014 to 2019 period (Figure 1A). Although rectal cancer had a higher risk of recurrence compared with colon cancer, a similar pattern of decreasing 5-year CIF was observed across the 3 periods: from 29.4% (95% CI, 27.8%-30.9%) in the 2004 to 2008 period, to 24.9% (95% CI, 23.4%-26.3%) in the 2009 to 2013 period, and to 18.3% (95% CI, 17.2%-19.5%) in the 2014 to 2019 period (Figure 1B). A consistent pattern of decreasing 5-year CIF of recurrence was also observed in stage-stratified analyses (eFigure 5 in Supplement 1). Tumor site– and stage-specific 1-, 3-, and 5-year CIFs of recurrence are presented in Table 2.

**Table 2.** Risk of Recurrence in Patients With Stages I to III Colorectal Cancer

Characteristic	Cumulative incidence function, % (95% CI)^a^			sHR (95% CI)^b^
	1 y	3 y	5 y	
**Colon cancer**				
UICC stage I				
2004-2008	3.4 (2.4-4.6)	11.5 (9.6-13.5)	16.3 (14.0-18.6)	1 [Reference]
2009-2013	4.1 (3.1-5.4)	9.5 (7.9-11.3)	12.5 (10.7-14.5)	0.75 (0.60-0.94)
2014-2019	1.5 (1.1-2.0)	4.4 (3.7-5.2)	6.8 (5.9-7.8)	0.37 (0.30-0.45)
UICC stage II				
2004-2008	7.2 (6.3-8.2)	17.0 (15.6-18.4)	21.9 (20.4-23.4)	1 [Reference]
2009-2013	5.4 (4.6-6.2)	13.6 (12.4-14.8)	17.0 (15.7-18.3)	0.76 (0.68-0.85)
2014-2019	3.6 (3.0-4.2)	9.7 (8.8-10.6)	11.6 (10.6-12.6)	0.50 (0.44-0.56)
UICC stage III				
2004-2008	12.2 (10.8-13.7)	29.8 (27.8-31.8)	35.3 (33.2-37.4)	1 [Reference]
2009-2013	11.6 (10.2-13.0)	26.9 (25.0-28.8)	31.1 (29.1-33.1)	0.85 (0.77-0.95)
2014-2019	9.8 (8.9-10.8)	21.8 (20.4-23.1)	24.6 (23.2-26.1)	0.65 (0.59-0.71)
**Rectal cancer**				
UICC stage I				
2004-2008	5.4 (4.0-7.1)	13.9 (11.6-16.4)	19.9 (17.2-22.8)	1 [Reference]
2009-2013	5.1 (3.8-6.7)	13.1 (11.0-15.3)	17.1 (14.8-19.6)	0.84 (0.68-1.05)
2014-2019	2.7 (2.0-3.5)	7.3 (6.2-8.5)	9.5 (8.2-10.9)	0.44 (0.36-0.55)
UICC stage II				
2004-2008	8.2 (6.8-9.9)	20.8 (18.5-23.1)	25.8 (23.4-28.2)	1 [Reference]
2009-2013	7.6 (6.1-9.2)	18.9 (16.7-21.2)	22.7 (20.4-25.2)	0.87 (0.74-1.02)
2014-2019	5.1 (4.0-6.4)	14.8 (13.0-16.8)	18.4 (16.3-20.6)	0.66 (0.56-0.79)
UICC stage III				
2004-2008	12.4 (10.6-14.2)	32.3 (29.7-34.8)	38.7 (36.0-41.3)	1 [Reference]
2009-2013	11.9 (10.2-13.8)	28.1 (25.6-30.6)	32.6 (30.0-35.1)	0.83 (0.73-0.94)
2014-2019	11.1 (9.6-12.7)	24.0 (21.9-26.1)	28.8 (26.6-31.1)	0.70 (0.62-0.80)

Abbreviations: sHR, subdistribution hazard ratio; UICC, Union for International Cancer Control.

^a^ Cumulative incidence function of colorectal cancer recurrence treating death and second primary cancer (other than colorectal cancer or nonmelanoma skin cancer) as competing events.

^b^ sHRs calculated by Fine-Gray regression adjusted for age, sex, and Charlson Comorbidity Index score.

### Time From Surgery to Recurrence

The proportion of diagnosed recurrences was highest in the first 3 years after surgery in all 3 calendar periods (eFigure 6 in Supplement 1). Over the 3 calendar periods, the proportion of recurrences diagnosed within the first 3 years after surgery increased (2004-2008, 80.0%; 2009-2013, 82.7%; and 2014-2019, 85.4%). From 2004 to 2008, the distribution of recurrences was dominated by a peak at 1 year postoperatively, while the pattern shifted such that by the 2014 to 2019 period, there were marked peaks at both year 1 and year 3 postoperatively (eFigure 7 in Supplement 1).

**Figure 2.**
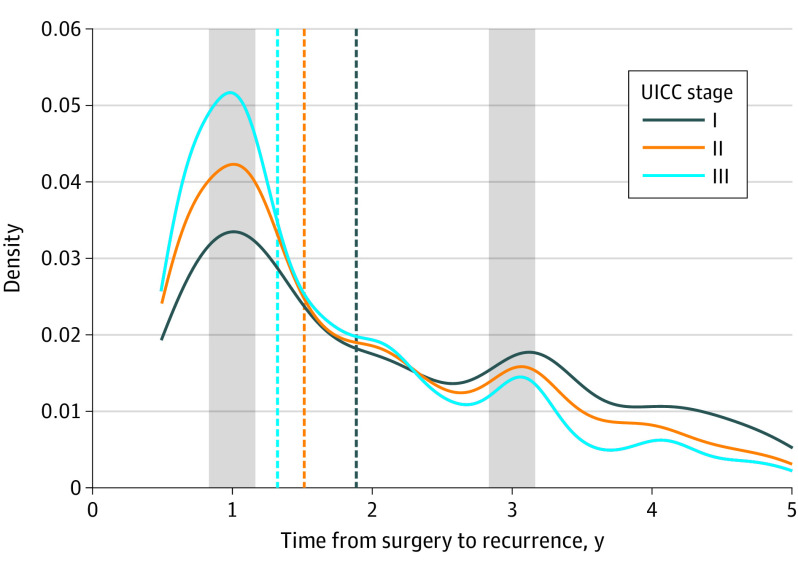
Time From Surgery to Colorectal Cancer Recurrence by Union for International Cancer Control (UICC) Stage Vertical dashed lines represent median time from curative surgery to recurrence. Shaded areas represent time points of surveillance imaging at 12 and 36 months after surgery (as per Danish guidelines since 2009).

Stage-specific TSTR revealed a shorter TSTR for patients with stage III CRC than for patients with stage II and particularly for those with stage I (Figure 2). The median (IQR) TSTR was 22.6 (20.2-24.2) months in patients with stage I CRC, 18.2 (17.2-19.1) months in those with stage II (time ratio stage II vs I, 0.58; 95% CI, 0.54-0.62), and 15.9 (15.4-16.5) months in those with stage III (time ratio stage III vs stage I, 0.30; 95% CI, 0.28-0.32). This difference was consistent through all 3 calendar periods (eFigure 8 in Supplement 1).

### Recurrences Associated With Screening Status

In the 2014 to 2019 period, 43.6% of patients with stage I CRC, 20.5% of those with stage II CRC, and 22.5% of those with stage III CRC were diagnosed through screening (eTable 4 in Supplement 1). Patients with screening-detected CRC more often had lower-stage disease and were more often male, were younger, and had fewer comorbidities at curative surgery compared with those with nonscreening-detected CRC.

**Figure 3.**
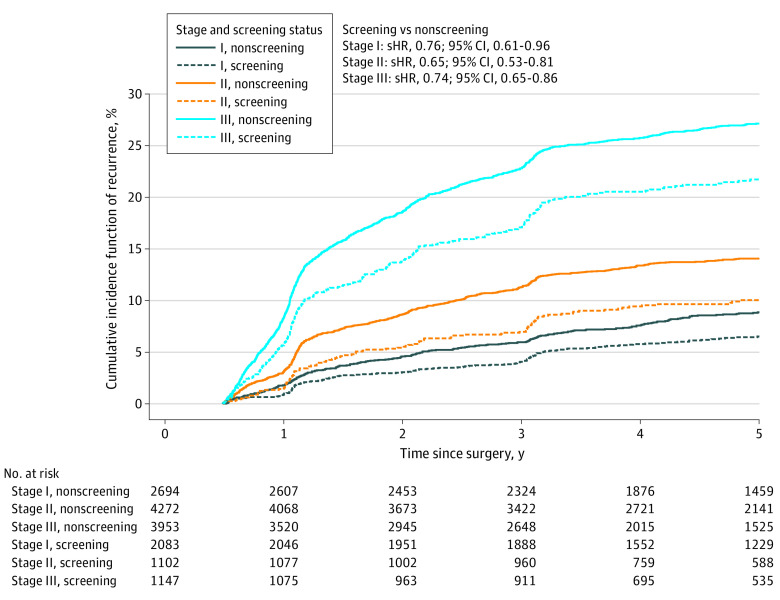
Cumulative Incidence Function Curves for Colorectal Cancer Recurrence According to Cancer Detection Method (Screening or Not Screening) Stratified by Union for International Cancer Control (UICC) Stage Curves were constructed using the Aalen-Johansen estimator. Patients were right-censored at emigration, 5 years of follow-up, or on January 1, 2023, whichever came first. sHR indicates subdistribution hazard ratio calculated using Fine-Gray regression adjusted for sex, age, comorbidities, and UICC stage.

Elaboration on the stage differences revealed that, within each N category, patients with screening-detected CRC had lower T categories than patients with nonscreening-detected CRC (eFigure 9 in Supplement 1). The stage-stratified 5-year CIF of recurrence was lower among patients with screening-detected CRC than in those with nonscreening-detected CRC (Figure 3; eTable 5 in Supplement 1). Screening-detected vs nonscreening-detected stage III CRC had a 5-year CIF of recurrence of 21.7% (95% CI, 19.3%-24.2%) vs 27.1% (95% CI, 25.7%-28.5%). The 5-year CIF of recurrence for screening-detected vs nonscreening-detected stage II CRC was 10.1% (95% CI, 8.3%-12.0%) vs 14.1% (95% CI, 13.0%-15.1%). The 5-year CIF of recurrence of screening-detected vs nonscreening-detected stage I CRC was 6.5% (95% CI, 5.5%-7.7%) vs 8.9% (95% CI, 7.8%-10.0%).

The risk of recurrence for screening-detected CRC was lower when adjusted for age, sex, comorbidity, tumor site, stage, and T category (sHR, 0.81; 95% CI, 0.73-0.91). For patients with nonscreening-detected CRC, the 5-year CIF of recurrence between 2014 and 2019 (eTable 5 in Supplement 1) was lower than the rates of recurrence prior to national screening implemented in 2014 (Table 2). Stage-specific reductions in recurrence were higher (by 0.8 to 5.6 percentage points) for patients with screening-detected CRC compared with nonscreening-detected CRC (eTable 6 in Supplement 1). Compared with nonscreening-detected CRC, the sHR was 0.76 (95% CI, 0.61-0.96) for stage I, 0.65 (95% CI, 0.53-0.81) for stage II, and 0.74 (95% CI, 0.65-0.86) for stage III screening-detected CRC.

## Discussion

From 2004 to 2019, many initiatives were implemented at a national level in Denmark to improve the treatment of patients with CRC (eFigure 1 in Supplement 1). This evolution of CRC management is not unique to Denmark, as international guidelines have changed multiple times over the past decades.^36,37,38,39^ While the combined implications of these initiatives have been associated with improved survival,^4,40^ their implications for risk of recurrence remain unexplored. The results of the present study show that the CRC recurrence rate has decreased substantially and continuously from 2004 to 2019, even after stratifying results by tumor site and UICC stage.

In 2014, population-based CRC screening via fecal immunochemical testing was implemented in Denmark and identified a group of younger patients with asymptomatic CRC. Consistent with reports from other countries,^19^ we saw that the screening prevalence round from 2014 to 2017 was associated with an increase in the number of patients with diagnosed CRC, with a shift toward lower UICC stages at diagnosis.^41^ Our comparison of screening-detected and nonscreening-detected CRC revealed lower rates of not only overall recurrence but also stage-specific recurrence in cancers detected by screening. Speculating whether screening was the main factor in the low stage-specific recurrence rates observed from 2014 to 2019, we explored the recurrence rate in patients whose cancer was not detected through screening. Their recurrence rate in the 2014 to 2019 period was still lower than recurrence rates observed in the 2 earlier periods, suggesting that other initiatives implemented in this period also had implications for the risk of recurrence (eFigure 1 in Supplement 1).

Additionally, detection through screening was associated with lower T category within each UICC stage. This change toward lower T category contributed to the lower stage-specific recurrence rates observed for the patients with screening-detected CRC, as advanced T category is a known factor associated with CRC recurrence.^42^ The T category–specific recurrence rates were also lower in patients with screening-detected vs nonscreening detected CRC. The persistent lower recurrence rate in those with screening-detected CRC could be due to a further downstaging of tumor burden beyond T category, such as tumor size, or to distinct differences in the biological characteristics, such as growth rate. Further studies are needed to address this finding.

The main goal of follow-up protocols is to detect recurrence early to maximize the patient’s survival. The European Society for Medical Oncology recommends a combination of clinical assessments, regular measurements of carcinoembryonic antigen levels, computed tomography (CT), and a colonoscopy with higher frequencies for the first 3 years after curative surgery and for a total duration of 5 years in patients with nonmetastatic CRC.^14,43^ However, substantial variation has been found between national surveillance guidelines,^44^ and a Cochrane Review^45^ found no benefit of intensified surveillance on overall survival. Denmark participated in the international multicenter randomized clinical trial COLOFOL,^46^ which did not show improved 5-year overall survival or CRC-specific survival associated with more frequent CT scans and carcinoembryonic antigen measurements (5 times vs 2 times), which is why Denmark follows a surveillance program with 2 CT scans.

We found that the 5-year CIF of recurrence is now below 7% and 10% for TNM stage I colon and rectal cancers, respectively (Table 2), and is even lower for cancers detected by screening. Furthermore, TSTR was longer for patients with stage I CRC than for patients with stage II and particularly stage III CRC. Due to this difference in the pattern of recurrence for patients with stage I disease, less intensive surveillance, or even no surveillance, may be noninferior to current guidelines—especially when also considering health-related quality of life, late sequelae, and cost-effectiveness.^47^ Interventional, preferably randomized studies are needed to explore shifting from one-size-fits-all surveillance toward a personalized surveillance strategy.^48^

### Strengths and Limitations

Strengths of the current study include the nationwide approach, reflecting standardized CRC management rather than the performance of individual centers. Also, the large sample size increased the statistical power and confidence of the reported recurrence rates.

Current knowledge about CRC recurrence is based primarily on intensive follow-up of selected patient cohorts, such as those enrolled in clinical trials or cohorts from individual centers.^49^ Herein we used registry data to obtain recurrence status, which is an efficient and inexpensive approach to obtain follow-up data compared with medical record review, which is time and resource intensive. Furthermore, we recently validated the algorithm we used and found that it was highly accurate (sensitivity, 94%; specificity, 99%; positive predictive value, 94%; and negative predictive value, 99%) when using contemporary registry data,^31^ which is in line with previous validations on historical cohorts.^30^ Also, the registry data needed for the algorithm is available for the entire cohort, as all Danish citizens have unrestricted access to a public tax-supported health care system.^50^

It can be considered a limitation that we excluded approximately 15% of patients, particularly patients with previous cancers, as the algorithm cannot discern whether recurrence occurs due to one or the other cancer. Although this exclusion may have introduced selection bias and affected the generalizability of the study, we decided to exclude these patients to improve the internal validity and accuracy of recurrence rates. The algorithm does not diagnose recurrence within 180 days after surgery. This quarantine was used to allow for completion of primary cancer treatment with up to 6 months of adjuvant chemotherapy and to avoid diagnosing synchronous metastases as a recurrence.^51^ Also, patients who died within 180 days after surgery were excluded to ensure all patients experienced time at risk of recurrence after 180 days.

## Conclusions

This register-based cohort study found substantial reductions in CRC recurrence risk for Danish patients with UICC stages I to III CRC from 2004 to 2019. The risk reductions were seen for all stages and for both colon and rectal cancers; reductions were especially notable in patients with screening-detected CRC but were also seen in patients whose CRC was not diagnosed through screening. Time to recurrence differed according to UICC stage. We believe that the risk of CRC recurrence has become so low in selected patient groups that further research on personalized surveillance protocols is indicated.

## Notes

Supplement 1. **eFigure 1.** Timeline of the Management of CRC in Denmark, 2004 to 2019 **eMethods.** Management of Nonmetastatic CRC in Denmark, 2004 to 2019 **eTable 1.** Dates for Last Update of the Included Registries and Their Tables **eFigure 2.** Algorithm Used to Identify CRC Recurrence from Danish Health Care Registries **eTable 2.** Classification Codes Used to Identify Oncological Departments From 2004 to 2022 **eFigure 3.** Flowchart of Cohort Selection **eResults.** Study Population **eFigure 4.** Distribution of UICC Stage I to III CRC From 2004 to 2019 by Tumor Site **eTable 3.** Multivariable Analysis Model of the Association Between Calendar Period of Primary Surgery and the Risk of Recurrence Within 5 Years After Primary Surgery in Stage I to III CRC **eFigure 5.** Cumulative Incidence Curves for CRC Recurrence by Calendar Period of Primary Surgery and Stratified by Tumor Site and UICC Stage **eFigure 6.** CRC Recurrences by Postoperative Year Stratified by Calendar Period of Primary Surgery **eFigure 7.** Density Plot of Time From Surgery to Recurrence in Patients with CRC Recurrence by Calendar Period **eFigure 8.** Time From Surgery to Recurrence (Among Patients With Recurrence Within 5 Years After Primary Surgery) by UICC TNM Stage in Patients with UICC Stage I to III CRC Treated in 2004-2008, 2009-2013, and 2014-2019 **eTable 4.** Patient Demographics and Treatment Characteristics of the 2014-2019 Study Population by Screening Status **eFigure 9.** Association Between TN Category and Screening Status in Patients with UICC Stage I to III CRC **eTable 5.** Rates and Hazards of Recurrence in Screening- vs Nonscreening–Detected UICC Stage I to III CRC, 2014 to 2019 **eTable 6.** Decrease in Risk of Recurrence by Screening Status and Improved Treatment from 2009-2013 and 2014-2019 in UICC Stage I to III CRC

Supplement 2.
Data Sharing Statement
