# Supplementary material for: Incidence of Recurrence and Time to Recurrence in Stage I to III Colorectal Cancer: A Nationwide Danish Cohort Study
Source: JAMA Oncol. 2023 Nov 16;10(1):54–62. doi: 10.1001/jamaoncol.2023.5098 (PMC10654928; doi:10.1001/jamaoncol.2023.5098)
Supplement: Supplement 1. — eFigure 1. Timeline of the Management of CRC in Denmark, 2004 to 2019 eMethods. Management of Nonmetastatic CRC in Denmark, 2004 to 2019 eTable 1. Dates for Last Update of the Included Registries and Their Tables eFigure 2. Algorithm Used to Identify CRC Recurrence from Danish Health Care Registries eTable 2. Classification Codes Used to Identify Oncological Departments From 2004 to 2022 eFigure 3. Flowchart of Cohort Selection eResults. Study Population eFigure 4. Distribution of UICC Stage I to III CRC From 2004 to 2019 by Tumor Site eTable 3. Multivariable Analysis Model of the Association Between Calendar Period of Primary Surgery and the Risk of Recurrence Within 5 Years After Primary Surgery in Stage I to III CRC eFigure 5. Cumulative Incidence Curves for CRC Recurrence by Calendar Period of Primary Surgery and Stratified by Tumor Site and UICC Stage eFigure 6. CRC Recurrences by Postoperative Year Stratified by Calendar Period of Primary Surgery eFigure 7. Density Plot of Time From Surgery to Recurrence in Patients with CRC Recurrence by Calendar Period eFigure 8. Time From Surgery to Recurrence (Among Patients With Recurrence Within 5 Years After Primary Surgery) by UICC TNM Stage in Patients with UICC Stage I to III CRC Treated in 2004-2008, 2009-2013, and 2014-2019 eTable 4. Patient Demographics and Treatment Characteristics of the 2014-2019 Study Population by Screening Status eFigure 9. Association Between TN Category and Screening Status in Patients with UICC Stage I to III CRC eTable 5. Rates and Hazards of Recurrence in Screening- vs Nonscreening–Detected UICC Stage I to III CRC, 2014 to 2019 eTable 6. Decrease in Risk of Recurrence by Screening Status and Improved Treatment from 2009-2013 and 2014-2019 in UICC Stage I to III CRC [file jamaoncol-e235098-s001.pdf]

Supplemental Online Content

Nors J, Iversen LH, Erichsen R, Gotschalck KA, Andersen CL. Incidence of recurrence and time to recurrence in stage I to III colorectal cancer, 2004 to 2019: a nationwide Danish cohort study. *JAMA Oncol.* Published online November 30, 2023. doi:10.1001/jamaoncol.2023.5098

eFigure 1. Timeline of the Management of CRC in Denmark, 2004 to 2019

eMethods. Management of Nonmetastatic CRC in Denmark, 2004 to 2019

eTable 1. Dates for Last Update of the Included Registries and Their Tables

eFigure 2. Algorithm Used to Identify CRC Recurrence from Danish Health Care Registries

eTable 2. Classification Codes Used to Identify Oncological Departments From 2004 to 2022

eFigure 3. Flowchart of Cohort Selection

eResults. Study Population

eFigure 4. Distribution of UICC Stage I to III CRC From 2004 to 2019 by Tumor Site

eTable 3. Multivariable Analysis Model of the Association Between Calendar Period of Primary Surgery and the Risk of Recurrence Within 5 Years After Primary Surgery in Stage I to III CRC

eFigure 5. Cumulative Incidence Curves for CRC Recurrence by Calendar Period of Primary Surgery and Stratified by Tumor Site and UICC Stage

eFigure 6. CRC Recurrences by Postoperative Year Stratified by Calendar Period of Primary Surgery

eFigure 7. Density Plot of Time From Surgery to Recurrence in Patients with CRC Recurrence by Calendar Period

eFigure 8. Time From Surgery to Recurrence (Among Patients With Recurrence Within 5 Years After Primary Surgery) by UICC TNM Stage in Patients with UICC Stage I to III CRC Treated in 2004-2008, 2009-2013, and 2014-2019

eTable 4. Patient Demographics and Treatment Characteristics of the 2014-2019 Study Population by Screening Status

eFigure 9. Association Between TN Category and Screening Status in Patients with UICC Stage I to III CRC

eTable 5. Rates and Hazards of Recurrence in Screening- vs. Nonscreening-Detected UICC Stage I to III CRC, 2014 to 2019

eTable 6. Decrease in Risk of Recurrence by Screening Status and Improved Treatment from 2009-2013 and 2014-2019 in UICC Stage I to III CRC

This supplemental material has been provided by the authors to give readers additional information about their work.

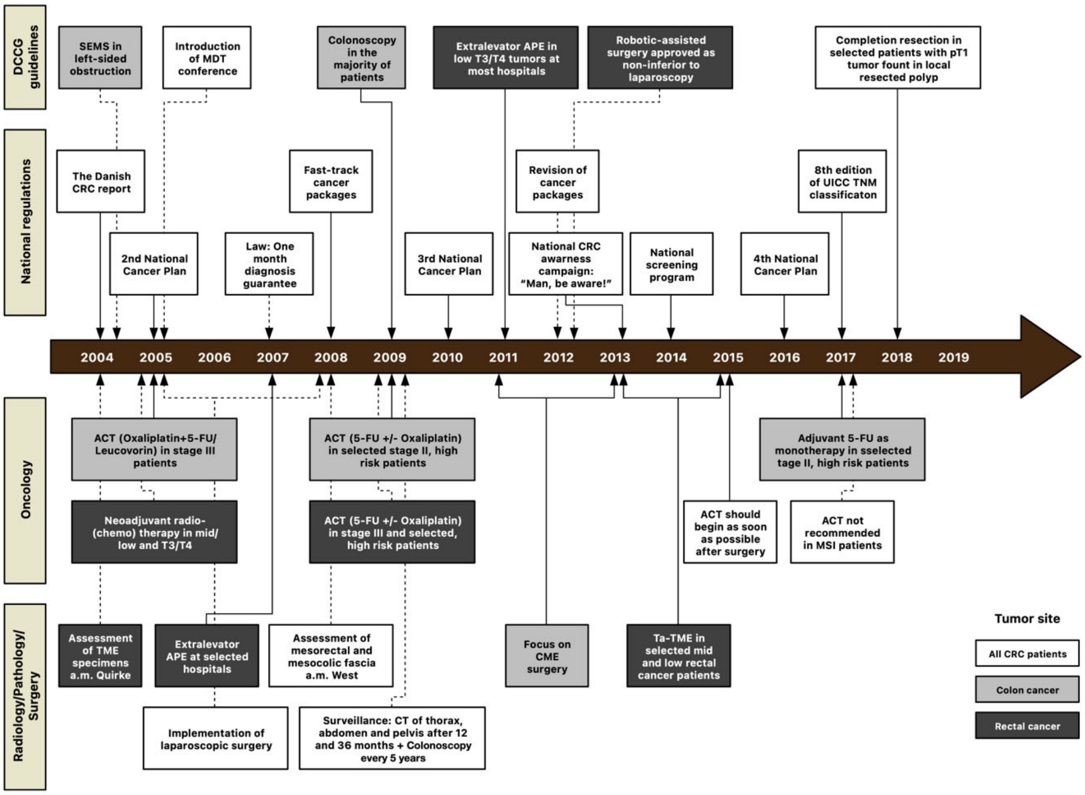

eFigure 1. Timeline of the Management of CRC in Denmark, 2004 to 2019

Timepoints are approximations.

Adopted from Iversen LH, Green A, Ingeholm P, Østerlind K, Gögenur I. Improved survival of colorectal cancer in Denmark during 2001-2012—the efforts of several national initiatives. *Acta Oncol.* 2016;55(suppl 2):10-23.).

eMethods: ***Management of Nonmetastastic CRC in Denmark, 2004 to 2019***

The diagnosis is based on histological verification. As per recommendations, patients receive a preoperative clinical TNM stage (cTNM) according to UICC classification of malignant tumors based on radiological imaging; CT of thorax, abdomen and pelvis and supplemented with MRI of the pelvis in rectal cancer patients. The treatment of all patients should be evaluated at a multidisciplinary team conference (MDT). Neoadjuvant treatment is guided by cTNM.

Following surgery with curative intent, the pathological TNM stage (pTNM) informs the use of adjuvant chemotherapy (ACT) for all stage III patients and stage II patients with clinical and histopathological risk factors. A complete colonoscopy to identify synchronous colorectal lesions is recommended postoperatively if not performed preoperatively.

All patients are offered postoperative surveillance that since 2009 includes CT scan of thorax, abdomen, and pelvis at 12 and 36 months, as a minimum, and colonoscopy every five years after surgery until age of 75 years.

The Danish CRC screening program was introduced in March 2014 and includes all individuals aged 50–74 years and is free of charge. Individuals are offered biennial screening using a fecal immunochemical test (FIT), and in case of a positive result (100 ng hemoglobin/ml), the individual is offered a colonoscopy.

Registry	Table(s)	Last update before data retrieval	Data retrieved
CRS	T_CIVIL	2022-12-21	2023-01-01
	T_CIVIL_HIST		
	T_PERSON		
	DANSK_OPHOLD_PERIODE_UNIK		
DCR	T_TUMOR	2022-03-25	2023-01-01
DPR	T_REKV_NY	2022-12-03	2023-01-01
	T_DIAGN_NY	2022-12-10	
DNPR	T_ADM	Not updated after the implementation of LPR3 in 2019	2023-01-01
	T_DIAG		
	T_SKSOPR		
	T_SKSUBE		
DNPR (LPR3)	DIAGNOSE	2022-12-31	2023-01-01
	KONTAKT		
	LOKALRECIDIVER		
	METASTASER		
DCCG	*NA*	2021-03-05	2021-03-05

eTable 1. Dates for Last Update of the Included Registries and Their Tables

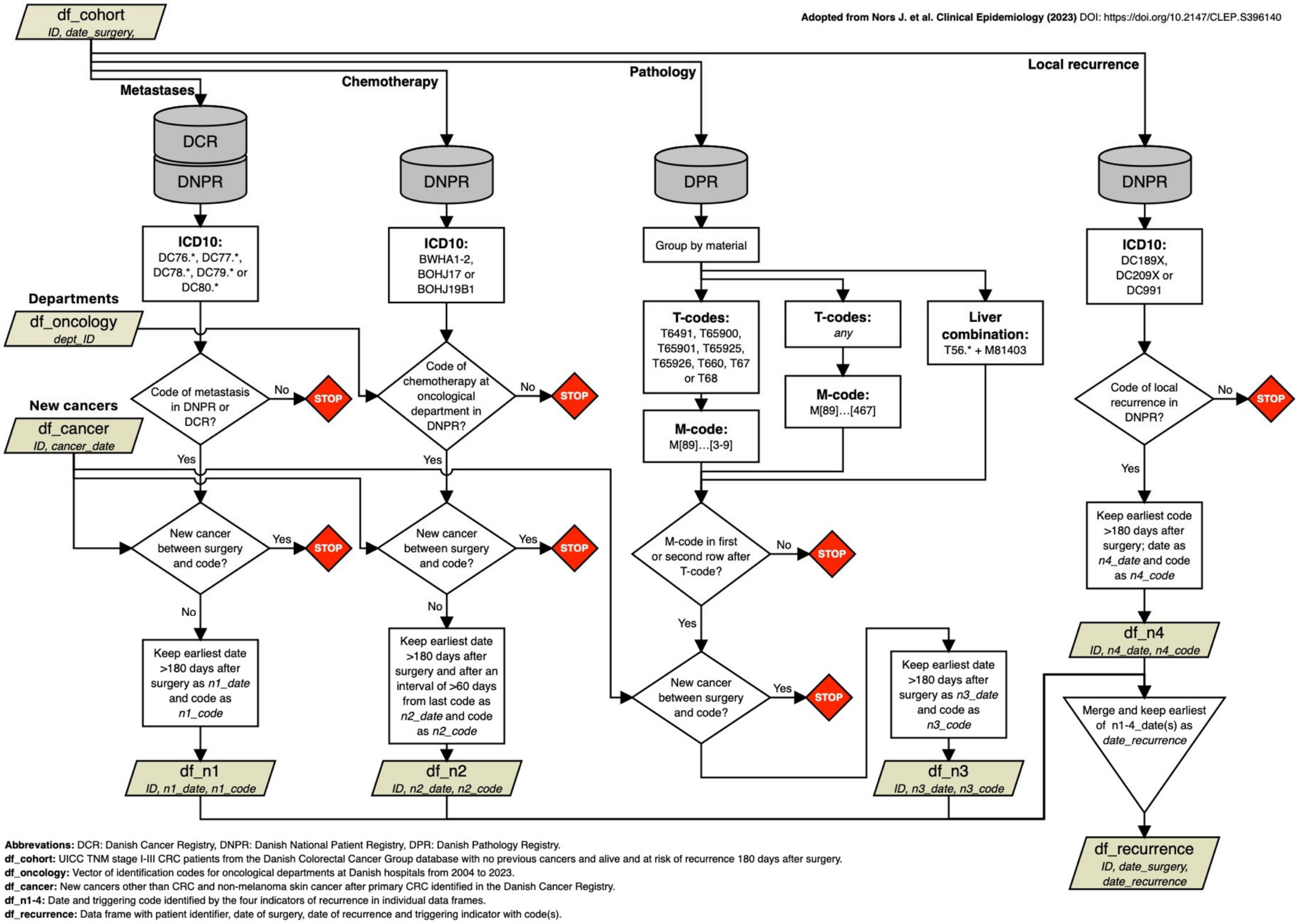

eFigure 2. Algorithm Used to Identify CRC Recurrence from Danish Health Care Registries

Modified from Nors J, Mattesen TB, Cronin-Fenton D, et al. Identifying recurrences among non-metastatic colorectal cancer patients using national health data registries: validation and optimization of a registry-based algorithm in a modern Danish cohort. *Clin Epidemiol.* 2023;15:241-250. Originally published by and used with permission from Dove Medical Press Ltd.

Registry	Hospital, City	Time Period	Classification Code
DNPR V2	Rigshospitalet, Copenhagen	2004-2019	130172.*
	Herlev Hospital, Herlev	2004-2019	151613.*
	Sjællands Universitetshospital, Roskilde	2007-2019	3800A9.*
		2004-2007	250119.*
	Næstved Sygehus, Næstved	2007-2019	3800N8.*
		2004-2007	350045.*|350120.*
	Bornholms Hospital, Rønne	2004-2019	400109.*
	Odense University Hospital, Odense	2004-2019	420226.*
	Sygehus Sønderjylland, Sønderborg/Aabenraa	2004-2019	5001057|500061.*
	Sydvestjysk Sygehus, Esbjerg	2005-2019	550105C|5501050
		2004	550105C|5501269
	Vejle Sygehus, Vejle	2004-2019	600818.*
	Hospitalsenheden Vest, Herning	2010-2019	665036.*
		2004-2012	650216.*
	Aarhus University Hospital, Aarhus	2004-2019	700325.*
		2011-2019	662025.*
	Aalborg University Hospital, Aalborg	2004-2019	800126:*
	Regionshospitalet Nordjylland, Hjørring	2006-2010	800326.*
DNPR V3	SPECIALE == “ONKOLOGI” (2019-2022)		

eTable 2. Classification Codes Used to Identify Oncological Departments From 2004 to 2022

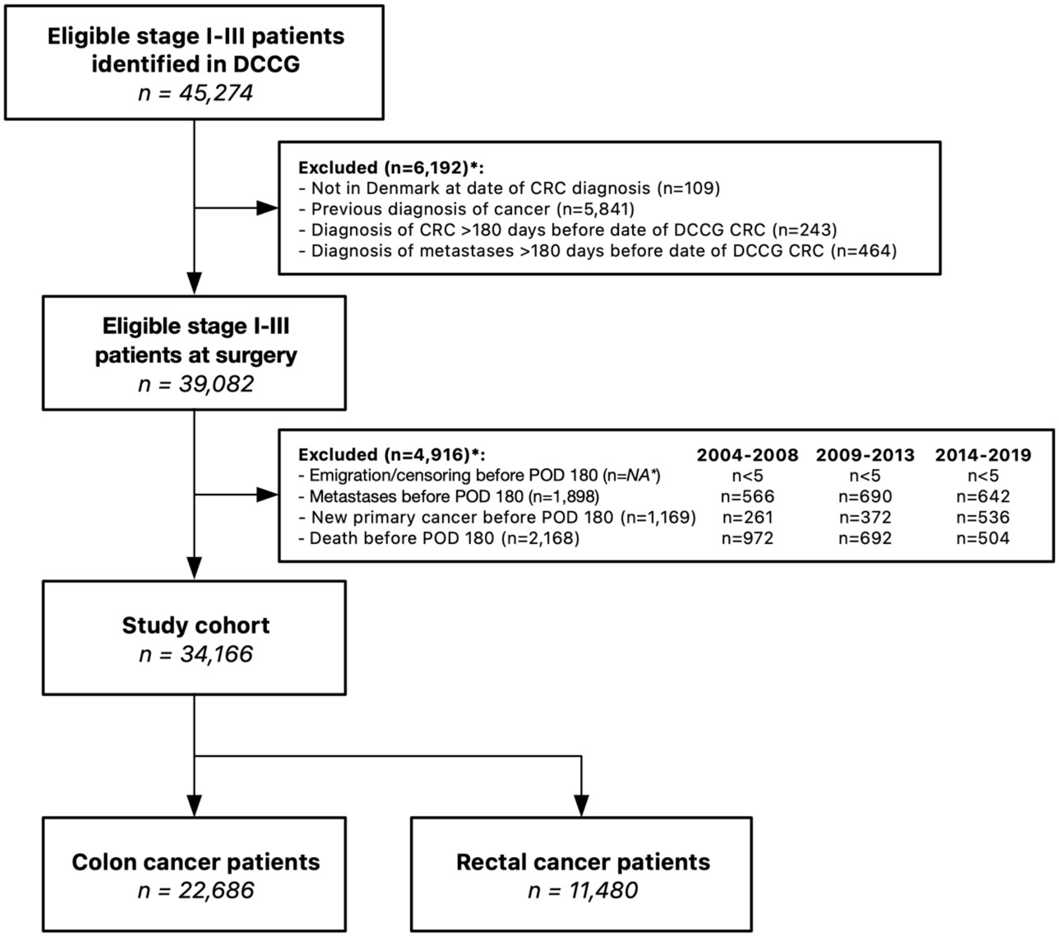

eFigure 3. Flowchart of Cohort Selection

All patients undergoing intended curative surgery for UICC TNM stage I-III CRC in Denmark from 1st January 2004, through 31st December 2019 were eligible for inclusion. Patients were excluded if they did not fulfill the requirements of the recurrence calling algorithm.

*Some patients were excluded due to more than one criterion, and cell-sizes <5 are reported in aggregate to reduce identification of individuals in the data.

Abbreviations: DCCG = Danish Colorectal Cancer Group. CRC = Colorectal cancer. POD = Postoperative day.

eResults: ***Study Population***

We identified 45,274 patients undergoing curative intent surgery for incident UICC TNM stage I-III CRC between January 1, 2004, and December 31, 2019 in the DCCG database. Initial exclusion involved 109 patient who were not in Denmark on date of CRC diagnosis according to the Danish Civil Registration System Registry (Danish: CPR-registret) and 5,841 with a history of a previous cancer other than CRC (ICD-10 DC18-20) or non-melanoma skin cancer (DC44). Furthermore, 243 were excluded due to diagnosis of CRC more than 180 days before CRC diagnosis in DCCG, and 464 patients were excluded due to diagnosis of metastases more than 180 days before CRC diagnosis in DCCG. Patients may be excluded due to more than one criterion.

This resulted in a cohort of 39,082 stage I-III patients, who were eligible at time of surgery. However, as the algorithm is not validated to identify recurrence within the first 180 postoperative days, as some patients receive 6 months of ACT and the M-category in DCR is updated retrospectively after 4 months (early metastases are interpreted as synchronous lesions and the patient is upstaged to UICC stage IV), we excluded 4,916 patients that either emigrated, were diagnosed with a second primary cancer, were diagnosed with metastases or died within the first 180 postoperative days. The proportion of patients excluded within 180 days from surgery decreased through the study period (2004-2008: 15.6%, 2009-2013: 14.5% and 2014-2019: 9.4%). This was primarily due to improved 180-day overall survival (2004-2008: 91.1% [95% CI: 90.5%-91.6%], 2009-2013: 94.0% [93.6%-94.5%] and 2014-2019: 97.0% [96.8%-97.3%]). The final cohort consisted of 34,166 patients, who all were at risk of recurrence 180 days after surgery.

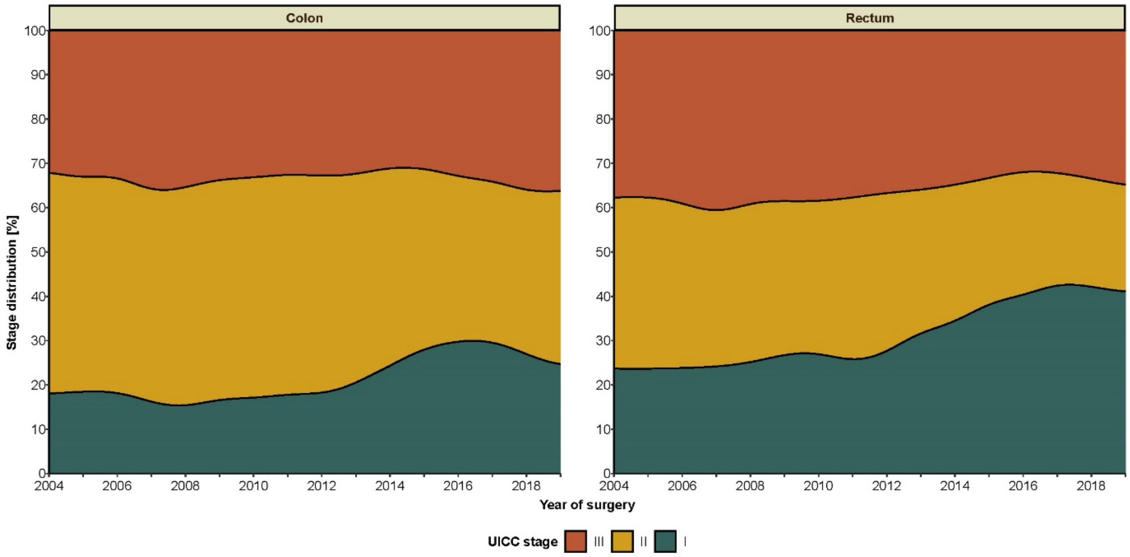

eFigure 4. Distribution of UICC Stage I to III CRC From 2004 to 2019 by Tumor Site

eTable 3. Multivariable Analysis Model of the Association Between Calendar Period of Primary Surgery and the Risk of Recurrence Within 5 Years After Primary Surgery in Stage I to III CRC

	Cumulative incidence function	Univariate regression		Multivariable regression	
**Characteristic**	**5-year CIF**	**sHR[1]**	**CI[1]**	**sHR[1]**	**CI[1]**
**Exposure variable**					
**Calendar period**					
2004-2008	27% (26%, 28%)	1.00	Reference	1.00	Reference
2009-2013	22% (22%, 23%)	0.80	0.76, 0.85	0.82	0.78, 0.87
2014-2019	16% (15%, 16%)	0.55	0.52, 0.58	0.59	0.56, 0.62
**Confounder variables**					
**Tumor site**					
Colon	19% (19%, 20%)	1.00	Reference	1.00	Reference
Rectum	24% (23%, 25%)	1.25	1.20, 1.32	1.26	1.20, 1.33
**UICC stage**					
I	12% (11%, 13%)	1.00	Reference	1.00	Reference
II	18% (17%, 19%)	1.60	1.49, 1.73	1.52	1.41, 1.64
III	31% (30%, 32%)	3.01	2.80, 3.22	2.80	2.61, 3.01
**Sex**					
Female	20% (19%, 20%)	1.00	Reference	1.00	Reference
Male	22% (21%, 23%)	1.14	1.09, 1.19	1.12	1.07, 1.18
**Age group at surgery**					
<55	25% (24%, 27%)	1.29	1.20, 1.39	1.18	1.10, 1.28
55-64	22% (21%, 23%)	1.09	1.02, 1.16	1.02	0.96, 1.09
65-74	21% (20%, 21%)	1.00	Reference	1.00	Reference
75-84	19% (19%, 20%)	0.93	0.87, 0.99	0.91	0.86, 0.97
>=85	16% (14%, 17%)	0.74	0.66, 0.83	0.72	0.64, 0.81
**Charlson Comorbidity Index**					
Comorbidity 0	21% (21%, 22%)	1.00	Reference	1.00	Reference
Comorbidity 1-2	20% (20%, 21%)	0.95	0.90, 1.00	1.05	1.00, 1.11
Comorbidity >3	19% (18%, 21%)	0.90	0.82, 0.98	1.06	0.97, 1.16
**Region of residence**					
Capital Region of Denmark	22% (21%, 23%)	1.00	Reference	1.00	Reference
Region of Northern Denmark	18% (17%, 19%)	0.81	0.74, 0.89	0.82	0.75, 0.89
Central Denmark Region	21% (20%, 22%)	0.98	0.91, 1.04	1.06	0.99, 1.13
Region of Southern Denmark	20% (19%, 21%)	0.92	0.86, 0.98	0.93	0.87, 1.00
Region Zealand	22% (21%, 23%)	1.00	0.93, 1.07	1.00	0.93, 1.07
**Surgical priority**					
Elective	20% (20%, 21%)	1.00	Reference	1.00	Reference
Emergency	33% (31%, 35%)	1.83	1.69, 1.98	1.69	1.55, 1.83

CIF: Cumulative incidence function of CRC recurrence treating death and second primary cancer as competing events. CIF considering each variable individually.

[1] sHR = Subdistribution hazard ratio by Fine-Gray regression, CI = Confidence Interval

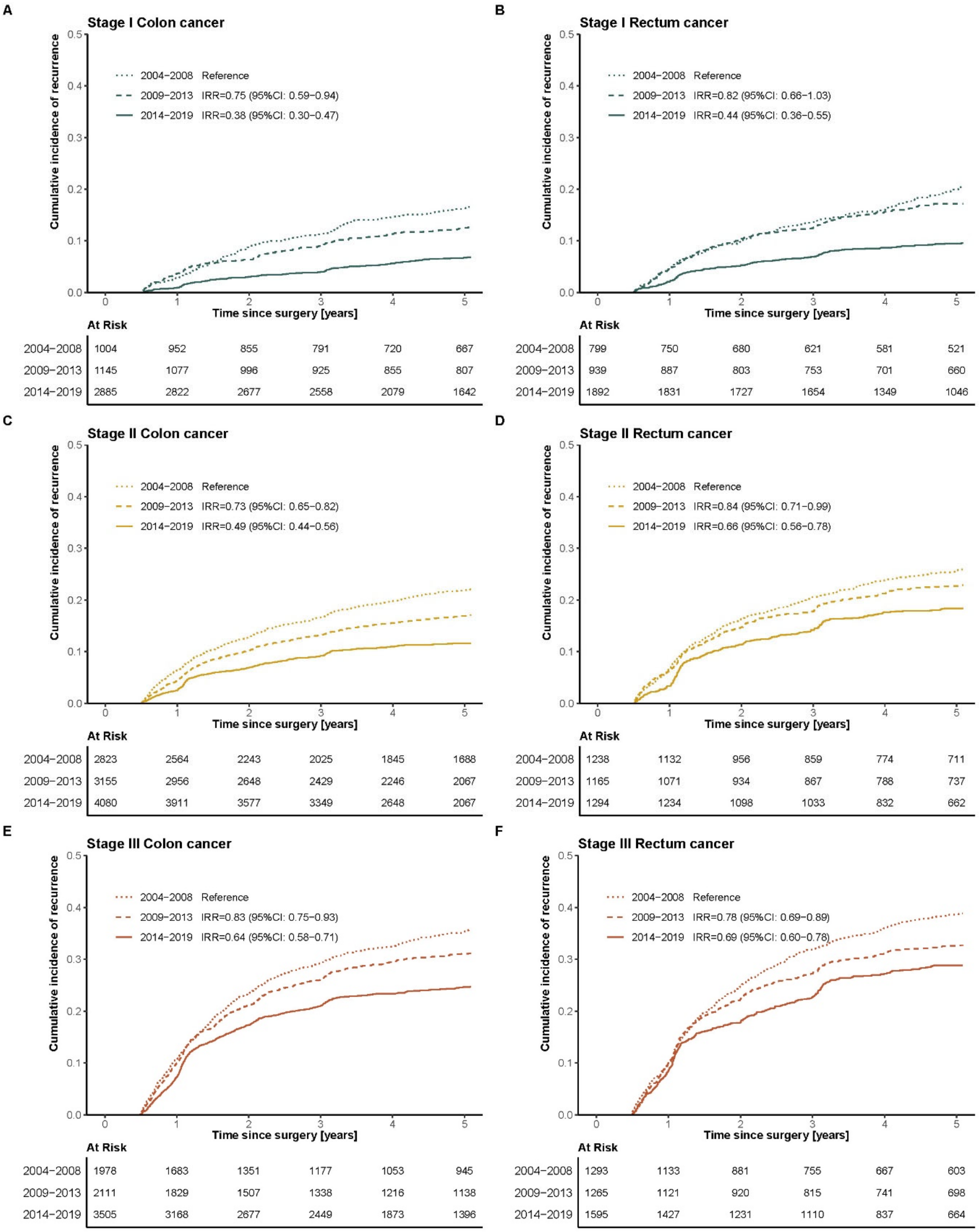

eFigure 5. Cumulative Incidence Curves for CRC Recurrence by Calendar Period of Primary Surgery and Stratified by Tumor Site and UICC Stage

Constructed using the Aalen-Johansen estimator. Patients were right-censored at emigration, five years of follow-up or on January 1st 2023.

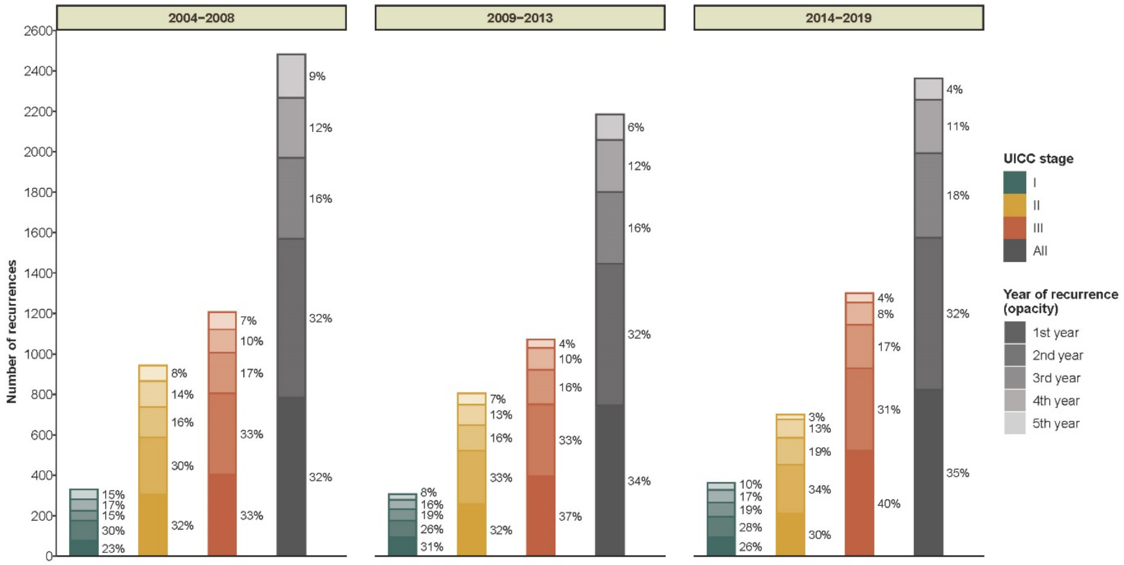

eFigure 6. CRC Recurrences by Postoperative Year Stratified by Calendar Period of Primary Surgery

Colored by UICC stage and opacity is determined by the time of recurrence. For each UICC stage we calculated the proportions by determining the number of recurrences diagnosed in each postoperative year and dividing it by the total number of recurrences within the calendar period.

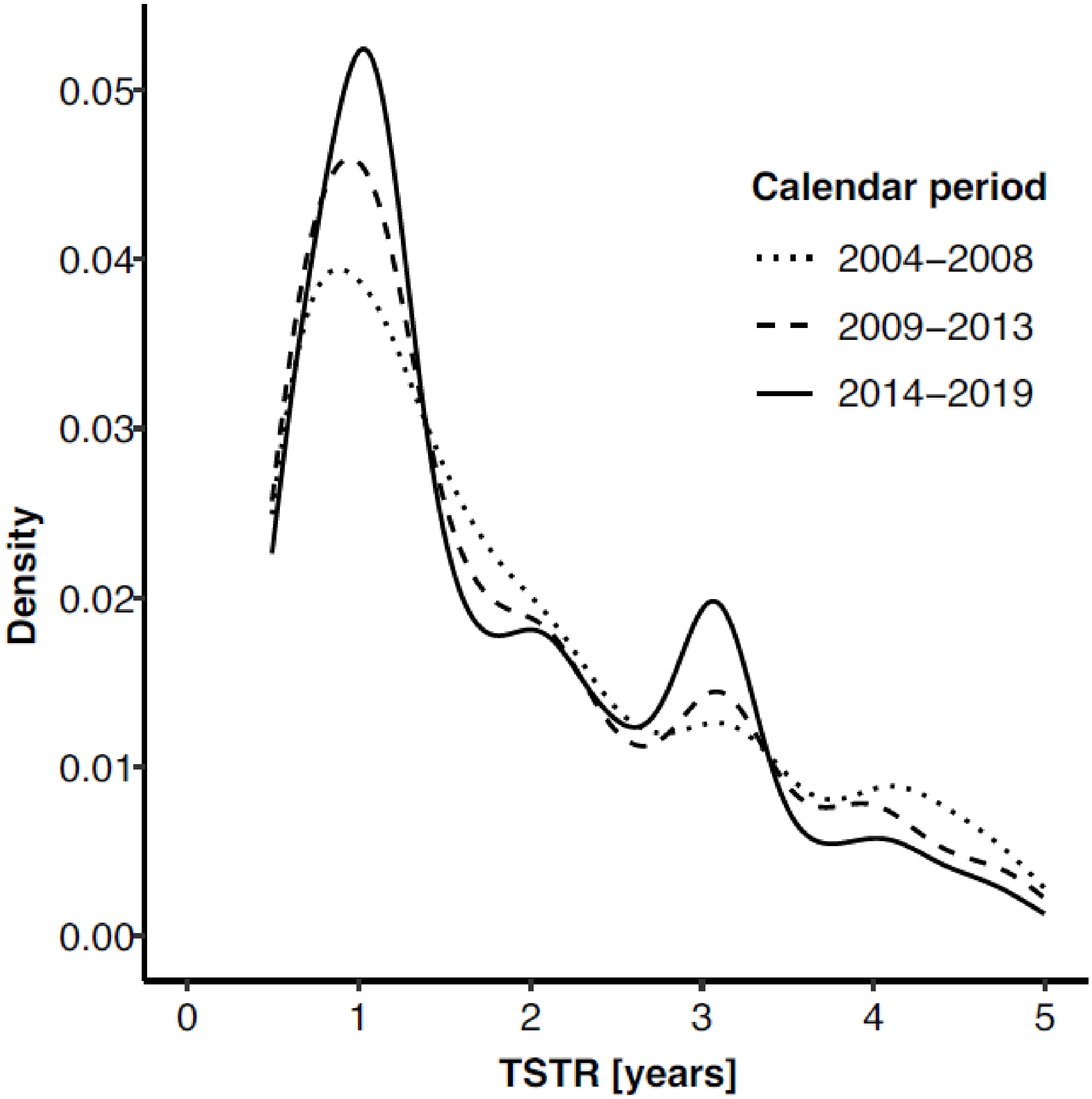

eFigure 7. Density Plot of Time From Surgery to Recurrence in Patients with CRC Recurrence by Calendar Period

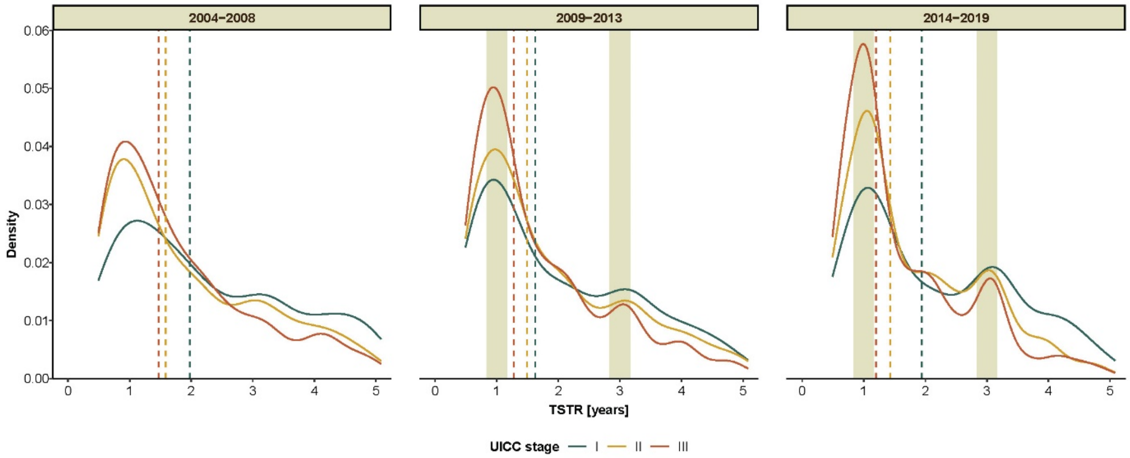

eFigure 8. Time From Surgery to Recurrence (Among Patients With Recurrence Within 5 Years After Primary Surgery) by UICC TNM Stage in Patients with UICC Stage I to III CRC Treated in 2004-2008, 2009-2013, and 2014-2019.

TSTR: Time from surgery to recurrence. Vertical lines represent median TSTR. Colored areas represent time points of surveillance imaging at 12 and 36 months (∓2 months) postoperative as per guidelines from 2009 and onwards.

Difference in TSTR between pUICC stages was estimated as time ratio (95% CI) using an accelerated failure time (AFT) model including tumor site, age, sex and Charlson Comorbidity Index score and right-censoring patients at second primary cancer (competing event), death (competing event), emigration, five years postoperative or January 1st 2023.

2004-2008 Time ratio$_{\text{stage II vs. I}}$ = 0.70 (95%CI: 0.62 – 0.78)

Time ratio$_{\text{stage III vs. I}}$ = 0.40 (95%CI: 0.35 – 0.45)

2009-2013 Time ratio$_{\text{stage II vs. I}}$ = 0.71 (95%CI: 0.62 – 0.81)

Time ratio$_{\text{stage III vs. I}}$ = 0.36 (95%CI: 0.32 – 0.41)

2014-2019 Time ratio$_{\text{stage II vs. I}}$ = 0.53 (95%CI: 0.47 – 0.59)

Time ratio$_{\text{stage III vs. I}}$ = 0.23 (95%CI: 0.20 – 0.26)

eTable 4. Patient Demographics and Treatment Characteristics of the 2014-2019 Study Population by Screening Status

Variable	Overall, N = 15,251[1]	Screening status NSD N=10,919	SD N=4,332
**Sex, n (%)**			
Female	6,821 (45%)	5,061 (46%)	1,760 (41%)
Male	8,430 (55%)	5,858 (54%)	2,572 (59%)
**Age, median (IQR)**	70 (63-76)	71 (63-79)	67 (62-72)
**Body mass index, median (IQR)**	25.8 (23.2-29.1)	25.5 (22.9-28.7)	26.6 (24.0-29.9)
**Charlson Comorbidity Index score, n (%)**			
Comorbidity 0	8,505 (56%)	5,977 (55%)	2,528 (58%)
Comorbidity 1-2	5,216 (34%)	3,744 (34%)	1,472 (34%)
Comorbidity >3	1,530 (10%)	1,198 (11%)	332 (7.7%)
**Place of residence, n (%)**			
Region of Northern Denmark	1,721 (11%)	1,221 (11%)	500 (12%)
Central Denmark Region	3,433 (23%)	2,479 (23%)	954 (22%)
Region of Southern Denmark	3,502 (23%)	2,481 (23%)	1,021 (24%)
Region Zealand	2,461 (16%)	1,692 (15%)	769 (18%)
Capital Region of Denmark	4,134 (27%)	3,046 (28%)	1,088 (25%)
**Tumor site, n (%)**			
Colon	10,470 (69%)	7,407 (68%)	3,063 (71%)
Rectum	4,781 (31%)	3,512 (32%)	1,269 (29%)
**UICC stage, n (%)**			
I	4,777 (31%)	2,694 (25%)	2,083 (48%)
II	5,374 (35%)	4,272 (39%)	1,102 (25%)
III	5,100 (33%)	3,953 (36%)	1,147 (26%)
**T-category*, n (%)**			
T1	2,513 (18%)	1,170 (12%)	1,343 (32%)
T2	2,642 (19%)	1,682 (17%)	960 (23%)
T3	6,800 (49%)	5,219 (53%)	1,581 (38%)
T4	1,673 (12%)	1,481 (15%)	192 (4.6%)
Tx	383 (2.7%)	284 (2.9%)	99 (2.4%)
**N-category*, n (%)**			
N0	8,728 (62%)	5,898 (60%)	2,830 (68%)
N1	2,969 (21%)	2,214 (23%)	755 (18%)
N2	1,488 (11%)	1,193 (12%)	295 (7.1%)
Nx	822 (5.9%)	529 (5.4%)	293 (7.0%)
**Adjuvant chemotherapy, n (%)**	4,203 (28%)	3,061 (28%)	1,142 (26%)
**Neoadjuvant treatment, n (%)**	1,240 (8.1%)	1,083 (9.9%)	157 (3.6%)

*Not reported for patients treated with neoadjuvant oncological therapy
NSD: non-screening detected, SD: screening detected

[1] n (%); Median (25%-75%)

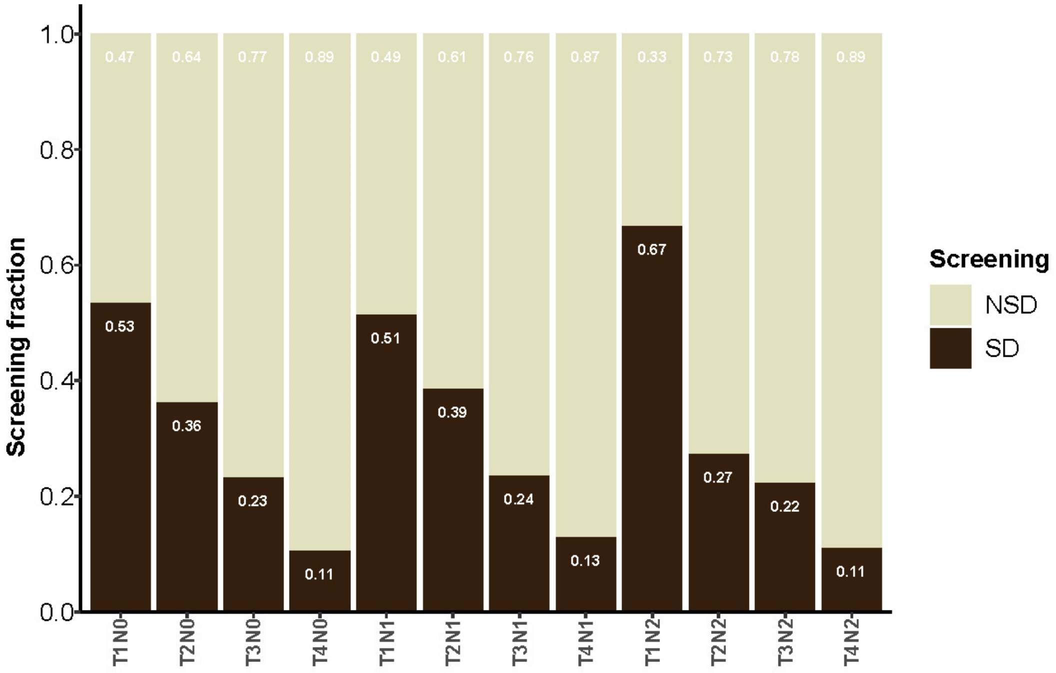

eFigure 9. Association Between TN Category and Screening Status in Patients with UICC Stage I to III CRC

NSD: non-screening detected. SD: screening detected.

eTable 5. Rates and Hazards of Recurrence in Screening- vs. Nonscreening-Detected UICC Stage I to III CRC, 2014 to 2019

	Cumulative incidence function			Fine-Gray regression	
**Characteristic**	**1-year CIF**	**3-year CIF**	**5-year CIF**	**sHR[1]**	**CI[1]**
		**Colon cancer**			
**UICC stage I**					
NSD	1.9% (95% CI: 1.3%-2.7%)	5.3% (95% CI: 4.2%-6.5%)	8.1% (95% CI: 6.8%-9.7%)	1.00	Reference
SD	1.0% (95% CI: 0.6%-1.6%)	3.4% (95% CI: 2.6%-4.5%)	5.3% (95% CI: 4.2%-6.6%)	0.65	0.47, 0.89
**UICC stage II**					
NSD	3.9% (95% CI: 3.2%-4.6%)	10.5% (95% CI: 9.5%-11.6%)	12.3% (95% CI: 11.2%-13.5%)	1.00	Reference
SD	2.4% (95% CI: 1.6%-3.6%)	6.7% (95% CI: 5.2%-8.5%)	8.9% (95% CI: 7.1%-10.9%)	0.64	0.49, 0.82
**UICC stage III**					
NSD	10.7% (95% CI: 9.6%-11.9%)	23.1% (95% CI: 21.5%-24.7%)	25.9% (95% CI: 24.2%-27.5%)	1.00	Reference
SD	6.8% (95% CI: 5.2%-8.7%)	17.3% (95% CI: 14.8%-20.0%)	20.3% (95% CI: 17.5%-23.2%)	0.71	0.59, 0.85
		**Rectum cancer**			
**UICC stage I**					
NSD	2.7% (95% CI: 1.9%-3.8%)	7.6% (95% CI: 6.2%-9.1%)	9.8% (95% CI: 8.2%-11.6%)	1.00	Reference
SD	2.6% (95% CI: 1.6%-4.0%)	6.9% (95% CI: 5.1%-8.9%)	9.0% (95% CI: 7.0%-11.3%)	0.90	0.65, 1.24
**UICC stage II**					
NSD	5.4% (95% CI: 4.1%-6.9%)	15.7% (95% CI: 13.5%-17.9%)	19.2% (95% CI: 16.9%-21.7%)	1.00	Reference
SD	3.9% (95% CI: 1.9%-6.9%)	11.1% (95% CI: 7.5%-15.5%)	14.5% (95% CI: 10.3%-19.5%)	0.68	0.47, 0.99
**UICC stage III**					
NSD	11.2% (95% CI: 9.6%-13.1%)	24.8% (95% CI: 22.5%-27.2%)	29.8% (95% CI: 27.3%-32.4%)	1.00	Reference
SD	10.6% (95% CI: 7.7%-14.1%)	20.9% (95% CI: 16.8%-25.4%)	25.0% (95% CI: 20.5%-29.7%)	0.82	0.64, 1.04

CIF: Cumulative incidence function of CRC recurrence treating death and second primary cancer (other than colorectal cancer or non-melanoma skin cancer) as competing event. NDS: non-screening detected, SD: screening detected.

[1] sHR = Subdistribution hazard ratio by Fine-Gray regression adjusted for age, sex and Charlson Comorbidity Index score, CI = Confidence Interval

eTable 6. Decrease in Risk of Recurrence by Screening and Improved Treatment from 2009-2013 and 2014-2019 in UICC Stage I to III CRC

		**2014-2019 Non-screening-detected CRC**		**2014-2019 Screening-detected CRC**		
**Characteristic**	**2009-2013 5-year CIF (95%CI)**	**5-year CIF (95%CI)**	**Percentage point reduction (NSD)**	**5-year CIF (95%CI)**	**Percentage point reduction (SD)**	**Additional reduction for screening-detected patients (SD-NSD)**
			**Colon cancer**			
**UICC stage I**	12.5% (10.7-14.5)	8.1% (6.8-9.7)	4.5%	5.3% (4.2-6.6)	7.2%	2.7%
**UICC stage II**	17.0% (15.7-18.3)	12.3% (11.2-13.5)	4,7%	8.9% (7.1-10.9)	8.1%	3.4%
**UICC stage III**	31.1% (29.1-33.1)	25.9 (24.2-27.5)	5.2%	20.3% (17.5-23.2)	10.8%	5.6%
			**Rectum cancer**			
**UICC stage I**	17.1% (14.8-19.6)	9.8% (8.2-11.6)	7.3%	9.0% (7.0-11.3)	8.1%	0.8%
**UICC stage II**	22.7% (20.4-25.2)	19.2% (16.9-21.7)	3.5%	14.5% (10.3-19.5)	8.2%	4.7%
**UICC stage III**	32.6% (30.0-35.1)	29.8% (27.3-32.4)	2.8%	25.0% (20.5-29.7)	7.6%	4.8%

CIF: Cumulative incidence function of CRC recurrence treating death and second primary cancer (other than colorectal cancer or non-melanoma skin cancer) as competing event.
95%CI: Confidence interval
CRC: Colorectal cancer, NSD: Non-screening detected, SD: Screening-detected.
