# Supplementary material for: Incidence of Recurrence and Time to Recurrence in Stage I to III Colorectal Cancer: A Nationwide Danish Cohort Study
Source: JAMA Oncol. 2023 Nov 16;10(1):54–62. doi: 10.1001/jamaoncol.2023.5098 (PMC10654928; doi:10.1001/jamaoncol.2023.5098)
Supplement: Supplement 2. — Data Sharing Statement [file jamaoncol-e235098-s002.pdf]

Data Sharing Statement

Nors. Incidence of Recurrence and Time to Recurrence in Stage I to III Colorectal Cancer. *JAMA Oncol*. Published November 30, 2023. doi:10.1001/jamaoncol.2023.5098

Data
Data available: No

Additional Information
Explanation for why data not available: The registry data used in this study is considered personal and protected patient data, in accordance with the Danish Data Protection act and the General Data Protection Regulations, and therefore cannot be disclosed. Data and material will not be available due to Danish Data Legislation.
